# Seasonal Baseline Evaluation of Physicochemical Parameters and Culturable Bacterial Characteristics in Northwestern Himalayan Ramsar Sites

**DOI:** 10.1111/1758-2229.70409

**Published:** 2026-09-01

**Authors:** Shagufta Butt, Chandresh Kumari, Rakesh Kumar Shukla, Sunil Puri, Sadanand Pandey, Adane Woldemedhin Kalsido

**Affiliations:** ^1^ School of Biological & Environmental Sciences, Shoolini University Solan Himachal Pradesh India; ^2^ Faculty of Applied Sciences and Biotechnology, Shoolini University Solan Himachal Pradesh India; ^3^ School of Physics and Materials Science, Faculty of Science, Shoolini University Solan Himachal Pradesh India; ^4^ Department of Hydraulic and Water Resources Engineering, College of Engineering and Technology Wachemo University Hosaina Ethiopia

**Keywords:** 16S rRNA, culturable bacterial isolates, culture‐dependent bacterial isolation, Ramsar wetlands, sanger sequencing, seasonal variation

## Abstract

Freshwater wetlands in the Himalayan region are ecologically sensitive and microbiologically diverse ecosystems, yet their seasonal dynamics remain insufficiently characterised. This study investigated seasonal variations in physicochemical properties and bacterial diversity of two Ramsar wetlands in northwestern India, Renuka Ji Lake and Asan Conservation Reserve. Water samples were collected during pre‐monsoon (June), monsoon (August) and post‐monsoon (November) seasons. Physicochemical parameters were analysed using standardised IS:3025, APHA 24th Edition and SLS/SOP protocols. Bacterial isolation was performed by serial dilution–pour plating on nutrient agar, followed by Gram staining, biochemical characterisation and 16S rRNA gene sequencing. Both wetlands exhibited pronounced seasonal fluctuations in water chemistry. At both sites, dissolved oxygen (Renuka Ji), turbidity and dissolved oxygen (Asan Conservation Reserve) exceeded CPCB permissible limits during specific seasons. Eleven bacterial isolates were identified, predominantly representing freshwater associated genera. *Agrobacterium cavarae* was comparatively uncommon in freshwater environments. No bacterial colonies were recovered from Renuka Ji Lake during post‐monsoon sampling within the tested dilution range (10^1^–10^4^). Overall, culturable bacterial communities showed seasonal variation and were dominated by a limited number of genera. These findings highlight the ecological sensitivity of Himalayan Ramsar wetlands and emphasise the need for continuous physicochemical and microbiological monitoring for effective conservation and sustainable management.

## Graphical abstract

Seasonal monitoring of two Himalayan Ramsar wetlands revealed significant fluctuations in physicochemical parameters and low bacterial diversity across seasons. Elevated dissolved oxygen at Renuka Ji, turbidity and dissolved oxygen at Asan Conservation Reserve levels highlight ecological sensitivity and the need for continuous monitoring and conservation of fragile freshwater ecosystems.

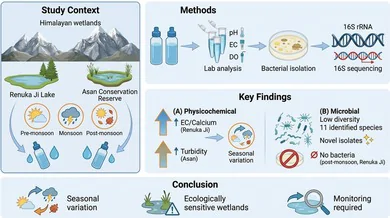

## 1 Introduction

Anthropogenic activities, climate variability and land‐use change are putting freshwater resources under increasing pressure. Wetlands, specifically, are at the centre of controlling the quality of water, facilitating biodiversity and mitigating ecological disruption (Mitsch and Gosselink 2015). Freshwater wetlands are particularly susceptible in high‐latitude areas and high‐altitude mountain slopes, where quick changes in land use worsen the dynamics of flood risk and interfere with natural hydrologic management (Dhakal et al. 2026), where physicochemical and microbiological processes are highly dependent on seasonal cycles of monsoon precipitation, snowmelt and temperature variation (Dhakar and Pandey 2020).

The Himalayan area of northern India contains several ecologically important wetland systems, two of which, Renuka Ji Lake and the Asan Conservation Reserve, were designated as Ramsar sites under the Ramsar Convention on Wetlands as an international wetland habitat and biodiversity. The two sites are located in the northwestern Himalaya and are geographically and hydrologically different, but experience comparable tourism, agricultural runoff and seasonal weather extremities. According to recent assessments, growing human stresses and agricultural runoff gradually change water quality indices (WQI) and hasten ecological degradation in local freshwater bodies (Pant et al. 2026).

The quality of water in these sensitive systems should be monitored with the evaluation of physicochemical parameters, including pH, electrical conductivity (EC), dissolved oxygen (DO), turbidity and nutrient load and microbiological indicators, including the structure and diversity of bacterial communities (WHO 2017; CPCB 2012). Bacteria are sensitive indicators of the health of the aquatic ecosystem and the seasonal changes in their abundance and richness are indicative of underlying changes in organic load, nutrient availability and physical conditions (Yang et al. 2024).

Despite their ecological value, detailed seasonal studies based on physicochemical and microbiological data in the Himalayan Ramsar sites are still limited. The combined seasonal trends of water chemistry and culturable bacterial populations have not been extensively studied; they mainly ignore the coupled, multi‐seasonal interaction between culturable bacterial communities and water chemistry, despite recent seasonal geochemical and water‐quality assessments elsewhere in the Himalayan region (Timalsina et al. 2025) and existing studies have focused on physicochemical profiling or short seasonal sampling windows (Sharma et al. 2018). By combining culture‐based techniques with molecular identification (16S rRNA sequencing), one can quantify and characterise culturable bacterial diversity by taxonomic traits, which gives a more comprehensive view of the microbial environment (Alsanie et al. 2018).

The current study was thus aimed at (i) determining the seasonal change in physicochemical parameters of Renuka Ji and Asan Conservation Reserve water samples under pre‐monsoon, monsoon and post‐monsoon seasons; (ii) isolating and characterising the culturable bacterial communities of both sites and (iii) molecularly identifying the bacterial isolates by the use of 16S rRNA gene sequencing. To our knowledge, this is the first study to combine seasonal physicochemical monitoring with culture‐based bacterial isolation and 16S rRNA identification at these two Ramsar sites.

## 2 Materials and Methods

### 2.1 Study Area and Sampling Sites

The sampling sites of the study were two Ramsar‐designated wetlands in the northwestern Himalaya of India, namely Renuka Ji Lake (Site 1) and Asan Conservation Reserve (Site 2).

Renuka Ji Lake is a natural lake located in Sirmaur District, Himachal Pradesh. It is the biggest natural lake in Himachal Pradesh and is of great religious, ecological and biodiversity importance. This site is a Ramsar wetland because it supports the migration of birds and also supports diverse flora and fauna. The geographical coordinates of the sampling area are between 30.609793° N and 30.610492° N latitude and 77.450088° E and 77.452640° E longitude.

Asan Conservation Reserve is situated at the confluence of the Yamuna and Asan rivers in the Dehradun District of Uttarakhand. It is a famous bird sanctuary and it was declared the first Ramsar site in Uttarakhand. The sampling coordinates are between 30.439453° N and 30.438534° N latitude and 77.669085° E and 77.668999° E longitude.

**FIGURE 1:**
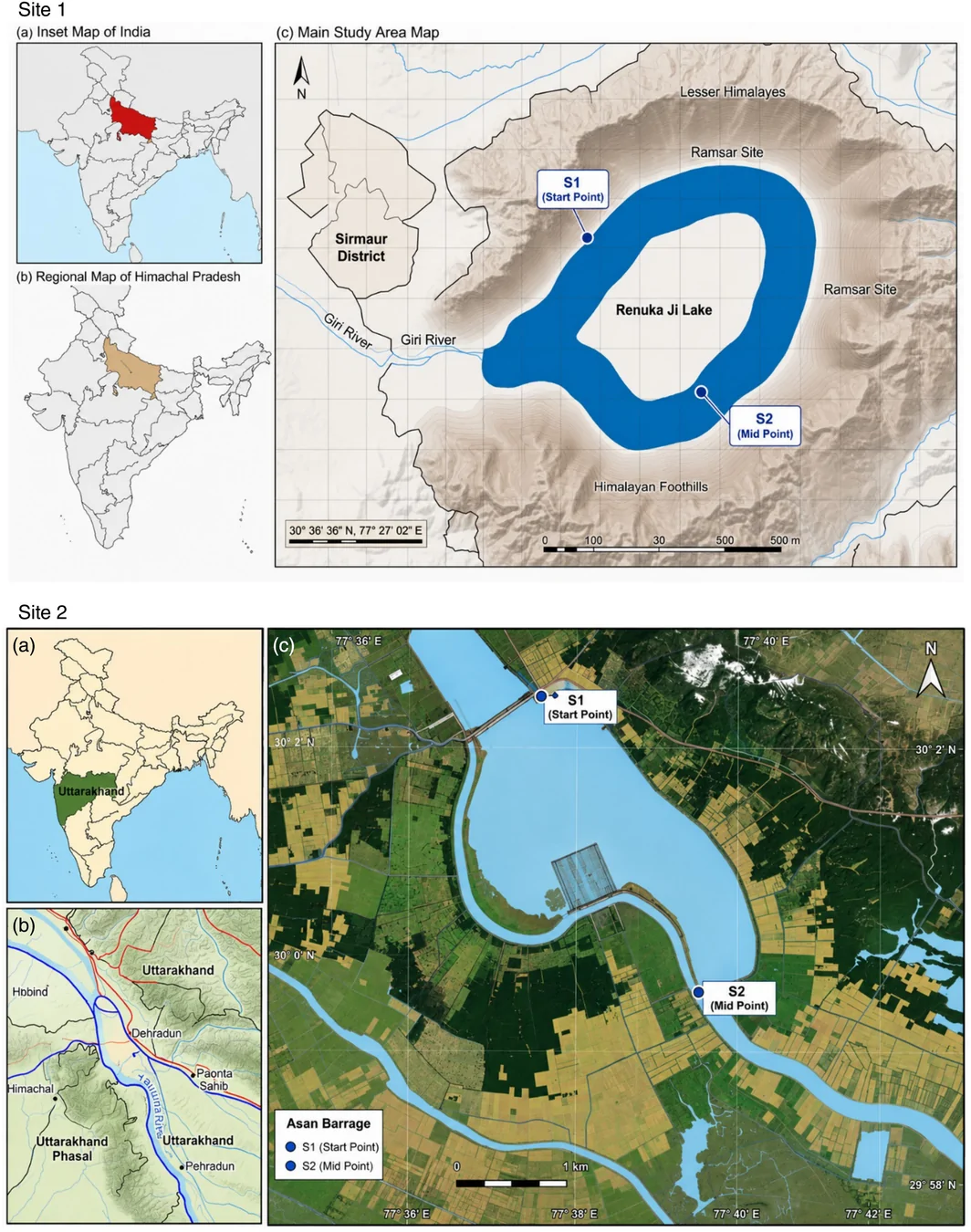
Location map of the two sampling sites: Renuka Ji Lake (Site 1) and Asan Conservation Reserve (Site 2), both Ramsar wetlands in the northwestern Himalaya, India, showing the water sampling points. (*Note:* Numerical datasets, statistical test details and *p*‐values are presented in the main text and Table 4 footnotes).

Both sites are indicative of ecologically sound Himalayan wetland systems, but they face different levels of anthropogenic stress. Figure 1 provides a location map of the two sampling sites.

### 2.2 Sample Collection

At each wetland, water samples were collected from 2 sampling points (i.e., starting point and midpoint) on 14/06/2024, 20/08/2024 and 20/11/2024, representing the pre‐monsoon, monsoon and post‐monsoon seasons of 2024. Water samples were collected in triplicate (*n* = 3). A total of 36 water samples (*n* = 18 per study site) were collected at each wetland at a depth of about 10 cm below the water surface, following standard surface water sampling procedures (APHA 2022). To avoid data repetition, additional sampling points were excluded due to the lake water's stagnant condition and thus limited spatial heterogeneity. Rather, in order to capture localised water quality characteristics and statistical consistency, 18 replicate samples were gathered at each site. All samples were placed in autoclaved, pre‐cleaned 1‐L high‐density polyethene (HDPE) bottles. A handheld GPS device was used to record the GPS coordinates of every sampling point. Sampling was done in three seasons: pre‐monsoon (June), monsoon (August) and post‐monsoon (November) to obtain the entire seasonal variation at each location.

Sampling was restricted to a single annual cycle; the seasonal patterns reported here therefore represent a seasonal snapshot rather than confirmed recurring dynamics and repeated multi‐year sampling would be needed to establish that inter‐seasonal differences are consistent from year to year.

### 2.3 Bacterial Isolation and Enumeration

Isolation for culturable heterotrophic bacteria, the serial dilution‐pour plate technique was used to isolate bacteria on nutrient agar (NA) medium. Water samples were diluted in sterile phosphate‐buffered saline (PBS) and proper dilutions were plated in triplicate. Plates were allowed to incubate at $37^{{}^{\circ}}C$ (24–48 h). The number of colony‐forming units per millilitre (CFU/mL) was counted and recorded on a seasonal and site basis. Incubation on NA at $37^{{}^{\circ}}C$ was used specifically to screen for culturable mesophilic, copiotrophic heterotrophs and possible terrestrial/anthropogenic microbial inputs to the wetland system during peak tourism and runoff seasons.

Colonies with morphologically different characteristics were chosen, subcultured and kept on NA slants to be further characterised. *Escherichia coli* (MTCC No. 3221) was taken as a reference control organism in case of biochemical testing and Gram staining and dilutions of 10^1^–10^4^ were plated in triplicate on NA following a standard serial dilution–pour plate procedure.

### 2.4 Gram Staining and Biochemical Characterisation

Gram staining was done using the standard four‐reagent protocol (crystal violet, Gram's iodine, acetone‐alcohol decolouriser and safranin counterstain) on all bacterial isolates. Biochemical tests were done based on the Manual of Systematic Bacteriology by Bergey (Goodfellow et al. 2012). The tests were the catalase test (hydrogen peroxide reaction), urease test (Christensen urea agar), citrate utilisation test (Simmons citrate agar), indole production test (tryptophan broth and Kovacs reagent) and motility test using semi‐solid agar. Assay‐specific positive and negative control reactions were used to maintain quality control (QC) for biochemical assays in compliance with standard diagnostic microbiology guidelines (CLSI 2024; Green and Goldman 2021). *E. coli* (MTCC No. 3221) served as the reference organism for catalase, indole and motility tests, for which it gives a positive reaction. *Klebsiella pneumoniae* served as the positive control for citrate utilisation and urease tests, for which *E. coli* is negative; *E. coli* itself served as the negative control for these two assays.

### 2.5 Chromosomal DNA Isolation

Chromosomal DNA was extracted from confirmed bacterial isolates according to the method of (Wright et al. 2017). In short, 5 mL of the overnight bacterial culture was centrifuged at 2200*g* for 5 min. The pellet was recentrifuged and washed with 1 mL TSE (Tris‐Saline‐EDTA) buffer. Five hundred microliter of TSE buffer containing lysozyme was added to the washed pellet and incubated at 37°C to digest the cell wall. The cell lysis was subsequently accomplished by the addition of 20 μL of proteinase K and 50 μL of 10% SDS, after which it was gently mixed and incubated at 60°C for 1 h. Cell lysate was moved into a clean tube and 500 μL phenol: chloroform (1:1) was added and mixed at top speed thoroughly for 15 min. The mixture was centrifuged at 10,000 rpm for 10 min and the top aqueous layer was transferred into a clean tube carefully. Phenol extraction was done twice to remove all the proteins. DNA precipitation was achieved by adding 50 μL of 3 M sodium acetate and 500 μL of isopropanol, mixing and centrifuging at 10,000 rpm for 15 min. The DNA pellet was rinsed in 0.5 mL of 70% ethanol, centrifuged at 10,000 rpm for 5 min and the ethanol was removed. The pellet was dried at room temperature and resuspended in 50–100 μL of TE buffer, then RNase was added and incubated at 37°C for 30 min to eliminate RNA contamination. The full stepwise workflow is provided in Figure S1.

### 2.6 Polymerase Chain Reaction (PCR) Amplification of 16S rRNA Gene

Universal primers were used to amplify the 16S rRNA gene from extracted chromosomal DNA. The PCR reaction was performed in a volume of 50 μL. Table 1 and Table 2 display the amplification conditions and the composition of the reaction mixture, whereas Table 3 contains details about the primers, following the primer design reported by (O'Sullivan et al. 2021).

**TABLE 1:** PCR thermal cycling conditions for 16S rRNA gene amplification.

Cycling conditions	Parameters	
Initial denaturation	3 min at 94°C	
Denaturation	1 min at 94°C	30 cycles
Annealing	1 min at 50°C	
Extension	2 min at 72°C	
Final extension	7 min at 72°C	

**TABLE 2:** PCR amplification reaction mixture composition.

PCR amplification conditions	Volume
DNA	1 μl
16S forward primer	2 μl
16S reverse primer	2 μl
dNTPs (2.5 mM each)	4 μL
10× Taq DNA polymerase assay buffer	5 μL
Taq DNA polymerase enzyme (3 U/μL)	1 μL
Water	35 μL
Total reaction volume	50 μL

**TABLE 3:** Primer details used for 16S rRNA gene amplification.

No.	Oligo name	Sequence (5′–3′)	*T*_m_ (°C)	GC content
1	16S forward	*GGATGAGCCCGCGGCCTA*	57	72.22%
2	16S reverse	*CGGTGTGTACAAGGCCCGG*	58	68.42%

The following are QC criteria for 16S rRNA gene amplification, controls and sequencing: A ~450 bp region (V9–V6 regions) of the 16S rRNA gene was amplified using primers 16S Forward (GGATGAGCCCGCGGCCTA) and 16S Reverse (CGGTGTGTACAAGGCCCGG) (Table 3). PCR was performed in a total volume of 50 μL, which contained 1 μL template DNA (160 ng), 2 μL of each primer (10 pmol/μL working stock; 0.4 μM final concentration), 4 μL dNTP mix (2.5 mM each), 5 μL 10× Taq assay buffer (1× final concentration, containing 3.2 mM MgCl_2_), 1 μL high‐fidelity DNA polymerase (3 U/μL) and 35 μL nuclease‐free water (Table 2).

Two control reactions were required in each PCR batch:

#### 2.6.1 No‐Template Control (NTC)

Nuclease‐free water instead of template DNA to show that the water is not contaminated with environmental or master mix reagent.

#### 2.6.2 Positive Control

To evaluate amplification efficiency and enzyme activities, genomic DNA from a verified reference strain of *E. coli* (ATCC 25922) was extracted.

The thermal cycling conditions are presented in Table 1. The integrity of amplicons was checked by running 5 μL of the PCR product on a 1.2% (w/v) agarose gel stained with ethidium bromide.

#### 2.6.3 Pre‐Sequencing Quality Criteria

A complete absence of amplification in the NTC, a single band with a sharp peak at the expected size of ~450 bp and a minimum DNA concentration of ≥ 20 ng/μL, were taken as strict criteria for proceeding with Sanger Sequencing. To quality‐filter chromatograms for bases with Phred scores *Q* ≥ 20 before contig assembly and BLASTn submission, the chromatograms were passed through a bi‐directional filter.

##### 2.6.3.1 PCR Conditions

*PCR amplification of 16S gene*:

160 ng of extracted DNA is used for amplification along with 10 pmol/μL of each primer.

*Composition of TAQ master MIX*:

1. High‐fidelity DNA polymerase
2. 2.5 mM dNTPs
3. 3.2 mM MgCl2
4. PCR enzyme buffer

To verify amplification, PCR products were separated on a 1.5% agarose gel. The purified amplified fragments were further sent to Biokart Pvt. Ltd., India, to undergo Sanger sequencing. The obtained nucleotide sequences were compared to identify similarities with the help of the BLAST (Basic Local Alignment Search Tool) software of the National Center for Biotechnology Information (NCBI). Confirmed sequences were entered into the NCBI GenBank database and accession numbers were received.

### 2.7 Physicochemical Analysis

Physicochemical analysis was performed at the Shoolini Life Sciences (SLS) Laboratory, Shoolini University, following Indian Standards (IS) methods and the laboratory's internal Standard Operating Procedures (SOP), as detailed below. Parameters examined were pH, EC, turbidity, total dissolved solids (TDS), total hardness, DO, biochemical oxygen demand (BOD), chemical oxygen demand (COD), calcium and chloride. The analysis procedures were pH—IS:3025 (P‐11): 2022; EC—SLS/SOP/CHE‐002; turbidity—IS:3025 (P‐10): 2023; TDS—IS:3025 (P‐16): 2023; total hardness—IS:3025 (P‐21): 2019; DO—SLS/SOP/CHE‐002; BOD—APHA 24th Edition, 5210B: 2022; COD—APHA 24th Edition, 5220B: 2022; calcium—SLS/SOP/ICP‐002 and chloride—IS:3025 (P‐32): 2003.

#### 2.7.1 Instrument Models

Calcium was determined using ICP‐OES (Thermo Scientific iCAP 7200). pH and EC of the samples were measured using LABMAN instruments, while DO and turbidity were measured using Systonic instruments. TDS was measured using a calibrated TDS/conductivity metre. Total hardness was determined by the EDTA titrimetric method. For BOD, DO was measured using a DO metre before and after incubation. COD was measured by dichromate reflux digestion followed by titration. Chloride concentration was determined by argentometric titration. Distilled water was used as the reagent blank throughout the analysis.

#### 2.7.2 Detection Limit

EC (0.1–20,000 μS/cm), Turbidity (0.1–1000 NTU), TDS (10–10,000 mg/L), total hardness (2–1000 mg/L), DO (1–20 mg/L), BOD (2–500 mg/L), COD (4–10,000 mg/L), calcium (0.5–100 mg/l), chloride (0.15–1000 mg/L).

### 2.8 Statistical Analysis

Data were analysed using SPSS and Microsoft Excel, with data processing and basic descriptive statistics. These data were tested for normality before analysis (Shapiro–Wilk test). The physicochemical parameters were normally distributed, so parametric data were used and results reported as the mean ± standard deviation (SD). The raw data tables are included in Tables S1–S3. Since additional comparative complexity tests were not required, summary data were presented as Table 4.

**TABLE 4:** Physicochemical properties of water samples during pre‐monsoon, monsoon and post‐monsoon at Renuka Ji (Site 1) and Asan Conservation Reserve (Site 2).

Parameter	Renuka Ji, pre‐monsoon	Renuka Ji, monsoon	Renuka Ji, post‐monsoon	Asan, pre‐monsoon	Asan, monsoon	Asan, post‐monsoon	Permissible limit
pH	8.28	8.55	8.33	6.53	7.99	6.54	6.5–8.5 (CPCB, Class D [propagation of wildlife and fisheries], 2012)
EC (μS/cm)	627.5	525.5	425	123.6	153.9	134.3	< 1000 μS/cm (CPCB, Class D guideline)
Turbidity (NTU)	4	5	5	22.0	197	16.7	1–5 NTU (BIS IS [Bureau of Indian Standards, Indian Standard] 10,500:2012)
TDS (mg/L)	315	269	302.5	110	80	105	500 mg/L (CPCB, Class A)
Total hardness (mg/L)	303	247	291	54	66	45	200–600 mg/L (BIS IS 10,500:2012)
DO (mg/L)	10	6.65	9.7	10.1	6.05	9.7	≥ 4.0 mg/L [CPCB, Class D (propagation of wildlife and fisheries], 2012)
BOD (mg/L)	10.65	14	16.95	12.3	9	19.95	30 mg/L (CPCB general standards for treated effluent discharge into inland surface waters, under the Environment Protection Rules, 1986 [General Statutory Rules 801(E)])
COD (mg/L)	117.3	144.8	104.8	134.65	152.65	92.95	250 mg/L (CPCB general standards for treated effluent discharge into inland surface waters, under the Environment Protection Rules, 1986 [General Statutory Rules 801(E)])
Calcium (mg/L)	94.092	66.96	93.2	61.46	46.61	39.31	75–200 mg/L (BIS IS 10,500:2012)
Chloride (mg/L)	5.40	5.44	4.10	3.34	2.24	1.53	250 mg/L (CPCB, Class A/C)

*Note:* The normality of data was checked by the Shapiro–Wilk test. All continuous variables were normally distributed (i.e., *p* > 0.05) except for turbidity for both sites. Mean ± standard deviations are shown in the Tables S1–S3.

## 3 Results

### 3.1 Physicochemical Parameters

At both sampling sites, physicochemical analysis was conducted in three seasons. The findings are presented in Table 4 for the case of pre‐monsoon, monsoon and post‐monsoon seasons, respectively.

The physical and mineral regimes at Renuka Ji (Site 1) and Asan Conservation Reserve (Site 2) are different in pre‐monsoon, monsoon and post‐monsoon seasons with common biological aeration patterns. Renuka Ji is consistently a moderately mineral‐dense wetland, alkaline (pH 8.28–8.55) and very clear with consistently low turbidity (< 5 NTU) and high TDS (269–315 mg/L), total hardness (247–303 mg/L) and EC (425–627.5 μS/cm) values. On the contrary, Asan Conservation Reserve has a less mineralised environment, being slightly acidic to near neutral (pH 6.53–7.99) outside the monsoon period and also has low conductivity (123.55–153.85 μS/cm), TDS (80–110 mg/L), total hardness (44–66 mg/L) and calcium (39.31–61.46 mg/L). However, turbidity is high throughout the year, with a mean turbidity of 16.7–21.95 NTU and can reach very high levels during the monsoon (up to 197 NTU) as a result of heavy surface runoff and inflow of suspended sediments. Contrasting these physical profiles, both ecosystems have a good level of oxygenation for sustaining aquatic life, with high levels of DO throughout the year, with a peak in pre‐monsoon (10 mg/L) and during post‐monsoon (9.7 mg/L), while a slight reduction is observed during monsoon (6.05–6.65 mg/L). In addition, both sites also have low chloride levels and stable oxygen demand parameters throughout all phases of the season, indicating that although seasonal rainwater runoff is a significant factor affecting clarity and mineral density, the overall organic and chemical load remains within safe limits for discharge to surface waters.

### 3.2 Bacterial Colony Counts

Enumeration of bacterial CFU/mL was done in serially diluted water samples on NA (Table 5). Unique seasonal and location‐specific patterns were observed.

**TABLE 5:** Bacterial colony counts (CFU/mL) from Renuka Ji and Asan Conservation Reserve across three seasons.

S. no.	Sampling site	Season	Colony count (CFU/mL)
1	Renuka Ji	Pre‐monsoon (June)	1.6 × 10^4^
2	Renuka Ji	Monsoon (August)	1.4 × 10^3^
3	Renuka Ji	Post‐monsoon (November)	Below detection limit
4	Asan Conservation Reserve	Pre‐monsoon (June)	2.51 × 10^4^
5	Asan Conservation Reserve	Monsoon (August)	1.96 × 10^3^
6	Asan Conservation Reserve	Post‐monsoon (November)	1.14 × 10^2^

*Note:* Bacterial counts were the highest in pre‐monsoon (1.6 × 10^4^ CFU/mL), followed by the monsoon (1.4 × 10^3^ CFU/mL) and no viable colonies were obtained/below detection limit in the post‐monsoon season. The maximum count was observed in pre‐monsoon (2.51 × 10^4^ CFU/mL), post‐monsoon (1.14 × 10^3^ CFU/mL) and the least in monsoon (1.96 × 10^2^ CFU/mL).

CFU/mL values were calculated from the dilution yielding 30–300 colonies per plate, plated in triplicate; where colonies were recovered at dilution (10^1^–10^4^), the result is reported as below the method detection limit rather than as a confirmed absence of bacteria.

Formula used to calculate CFU is:

$$\mathrm{CFU}/\mathrm{mL}=\frac{\text{Mean number of colonies}}{\text{Volume plated}\;\left(\mathrm{mL}\right)\times \text{Dilution factor}}$$

### 3.3 Gram Staining and Biochemical Characterisation

Thirteen bacterial isolates were identified by Gram staining and biochemical tests in all seasons and locations. Isolates that have been confirmed by NCBI molecular analysis are marked with an asterisk (*). The results of microbial identification by season and site are presented in Tables 6, 7, 8, 9.

**TABLE 6:** Microbial identification results of bacterial isolates during pre‐monsoon at Renuka Ji sampling site.

S. no.	Bacterial species	Type	Catalase test	Urease test	Citrate test	Indole test	Motility test
1	Endophytic bacterium*	Gram negative	+	+	+	+	Motile
2	*Pseudomonas baetica*	Gram negative	+	+	+	+	Motile
3	*Comamonas* sp.*	Gram negative	+	+	−	+	Motile
4	*Fictibacillus* sp.*	Gram positive	+	−	−	+	Motile

*Note:* Isolates that have been confirmed by NCBI molecular analysis are marked with an asterisk (*).

Abbreviations: + = positive reaction; − = negative reaction.

**TABLE 7:** Microbial identification results of bacterial isolates during pre‐monsoon at the Asan Conservation Reserve sampling site.

S. no.	Bacterial species	Type	Catalase test	Urease test	Citrate test	Indole test	Motility test
1	*Acinetobacter* sp.	Gram negative	+	+	+	−	Non‐motile
2	*Acinetobacter courvalinii*	Gram negative	+	+	+	+	Non‐motile

**TABLE 8:** Microbial identification results of bacterial isolates during the monsoon at Renuka Ji and Asan Conservation Reserve sampling sites.

S. no.	Bacterial species	Type	Catalase test	Urease test	Citrate test	Indole test	Motility test
1	*Acidovorax* sp.* (Renuka Ji)	Gram negative	−	−	−	+	Motile
2	Agrobacterium cavarae* (Asan Conservation Reserve)	Gram negative	+	+	+	+	Motile
3	Pseudomonas fulva* (Asan Conservation Reserve)	Gram negative	+	+	+	+	Motile

*Note:* Isolates that have been confirmed by NCBI molecular analysis are marked with an asterisk (*).

**TABLE 9:** Microbial identification results of bacterial isolates during the post‐monsoon at the Asan Conservation Reserve sampling site.

S. no.	Bacterial species	Type	Catalase test	Urease test	Citrate test	Indole test	Motility test
1	Planococcus citreus*	Gram positive	+	+	+	+	Motile
2	*Pseudomonas* sp.	Gram negative	+	+	+	+	Motile
3	Exiguobacterium undae*	Gram positive	−	−	−	−	Motile
4	Planococcus ruber*	Gram positive	+	+	−	+	Motile
5	Bacillus thuringiensis*	Gram positive	+	−	−	−	Motile
6	*Exiguobacterium* sp.*	Gram positive	+	−	−	−	Motile

*Note:* Species marked with * are molecularly verified newly reported isolates from these Ramsar‐protected Himalayan wetlands.

^a^ Molecularly verified newly reported isolates from these Ramsar‐protected Himalayan wetlands.

There are 9 Gram‐negative bacteria and 6 Gram‐positive bacteria; universal motility was observed in all 15 bacterial species, with evidence of active flagellar movement, which aids in motility towards root exudates and a microenvironment favourable for survival (Ahemad and Kibret 2014; Backer et al. 2018). The Gram‐negative group consists of opportunistic genera that are associated with plants ( *Pseudomonas baetica* , *P. fulva* , *Acidovorax* sp., *Comamonas* sp., *Agrobacterium cavarae*). The Gram‐positive isolates include endospore‐forming or stress‐tolerant environmental strains, like *Bacillus thuringiensis* , *Fictibacillus* sp., *Planococcus citreus* , *P. ruber* and *Exiguobacterium undae* .

### 3.4 Metabolic and Enzymatic Profiling

The enzymatic activity exhibits high adaptation to oxidative stress, nitrogen cycling and organic carbon utilisation.

#### 3.4.1 Catalase Activity

Most of the isolates break down hydrogen peroxide to water and oxygen to protect the cells from reactive oxygen species generated during aerobic respiration or host defence responses (Mamarasulov et al. 2022; Hanaoka et al. 2013). *Acidovorax* sp. and *E. undae* were the only two strains that were catalase negative.

#### 3.4.2 Indole Production

Most isolates are positive for tryptophanase activity that produces indole from tryptophan. Production of indole is one of the well‐known traits of phytostimulatory activity, since indole compounds are the precursors for auxin biosynthesis (e.g., Indole‐3‐Acetic Acid), which stimulate root elongation and plant growth (Ahemad and Kibret 2014; Shah et al. 2022).

#### 3.4.3 Urease Activity

Over half of the tested species are urease positive, which means that they convert urea into ammonia and carbon dioxide. This ureolytic activity leads to more mineralised nitrogen in agricultural soil, which is more available for plant root uptake (Backer et al. 2018; Salo and Novero 2020).

#### 3.4.4 Citrate Versatility

The versatility in the capacity of the isolates to use citrate as a sole carbon source and energy source was restricted to six isolates (*P. citreus, A. cavarae, Pseudomonas* sp. and *Endophytic bacterium*) that possess citrate permease (Mamarasulov et al. 2022).

### 3.5 Functional and Ecological Clustering

The biochemical characteristics place the 15 bacteria into three functional ecological niches.

#### 3.5.1 Plant Growth‐Promoting Endophytes and Rhizobacteria (PGPR/PGPE)

Cluster of isolates containing *A. cavarae, Pseudomonas fulva* and *P. baetica* shows positive results in all 5 biochemical tests. This metabolic fusion allows for the active colonisation of the roots, nitrogen transformation and the secretion of phytohormones (Ahemad and Kibret 2014; Shah et al. 2022).

#### 3.5.2 Biocontrol and Environmental Remediation Agents

Species like *B. thuringiensis, Comamonas* sp. and *Acidovorax* sp. exhibit special metabolic patterns. *B. thuringiensis* is motile and catalase‐positive, but urease‐ and citrate‐negative and is therefore oxidative, but not ureolytic and citrate‐negative. The genera *Comamonas* and *Acidovorax* are important genera that degrade organic contaminants in aquatic and soil environments (Backer et al. 2018).

#### 3.5.3 Extremophilic and Stress‐Tolerant Taxa

*P. citreus* , *P. ruber* and *E. undae* are known for their saline, thermal and cold stress tolerance, respectively. Although *P. citreus* is very metabolically versatile, the metabolic biochemical profile of *E. undae* is very limited, suggesting that the metabolic pathways are optimised to cope with extreme environmental stress, not with fast carbohydrate turnover (Hanaoka et al. 2013).

### 3.6 Molecular Identification and NCBI Accession Numbers

A total of 11 bacterial isolates were obtained by 16S rRNA gene sequencing using the Sanger Sequencing technique at Biokart Pvt. Ltd., India. The resulting sequences were uploaded to NCBI GenBank and were given accession numbers (Table 10). Similarities between the nucleotides were evaluated with the help of the BLAST software of NCBI. The 11 isolates are all important bacterial taxa that were obtained in these Himalayan Ramsar wetlands and some of them are potentially new records in these environments.

**TABLE 10:** List of bacterial isolates with strain numbers and NCBI GenBank accession numbers.

S. no.	Name of bacteria	Strain number	NCBI accession number
1	*Fictibacillus* sp.	AK57	PX980612.1
2	*Comamonas* sp.	20‐RIF‐16	PX980613.1
3	*Endophytic bacterium*	GGR	PX980614.1
4	*Acidovorax* sp.	C9‐1	PX980615.1
5	*Pseudomonas fulva*	2–5	PX980616.1
6	*Agrobacterium cavarae*	RZME10	PX980617.1
7	*Planococcus citreus*	ZBN‐491	PX980618.1
8	*Exiguobacterium undae*	B111	PX980619.1
9	*Planococcus ruber*	CD8	PX980620.1
10	*Exiguobacterium* sp.	WY(Y)3	PX980621.1
11	*Bacillus thuringiensis*	S8‐3_EP	PX980622.1

*Note:* Taxonomic assignments reflect the resolution achieved by 16S rRNA BLAST comparison; isolates designated with ‘sp.’ represent genus‐level identification and ’Endophytic bacterium’ represents provisional unclassified assignments based on top match against reference strain database; entries with ‘sp.’ indicate that species‐level identity could not be confidently resolved from partial 16S sequence data alone.

For each isolate, taxonomic assignment was based on ≥95% sequence identities ranging between 95% and 100% query coverage against the closest GenBank match (*E*‐value 0.0), using BLASTn; NCBI. A neighbour‐joining phylogenetic tree with 1000 bootstrap replicates. A comprehensive Table S4 containing all BLAST parameters, including query coverage, percent identity, *E*‐values, accession numbers and top matches for all 11 isolates.

## 4 Discussion

### 4.1 Seasonal Variation in Physicochemical Parameters

The physicochemical data from the two Ramsar wetland sites showed strong seasonal fluctuations. The alkaline pH at Renuka Ji throughout all three seasons (8.28–8.55) could be explained by the calcareous geology of the watershed, the photosynthetic activity of the aquatic macrophytes and the decreased dilution in drier months (Das and Kaur 2008; Kumar et al. 2019). By comparison, the Asan Conservation Reserve exhibited almost neutral to slightly acidic pH, which is in line with its riverine and estuarine nature and the effects of the Himalayan River runoff.

EC at Renuka Ji in all seasons the maximum EC was observed in pre‐monsoon (627.5 μS/cm). It is probably associated with the fact that the dissolved salts and calcium carbonate compounds of the surrounding geological formations are concentrated in low‐flow conditions (Bala et al. 2017). The dilution effect of monsoon rainfall was observed in the low values of EC that were recorded during the monsoon season at both sites.

The turbidity of Asan Conservation Reserve increased rapidly during the monsoon (197 NTU), which is significantly higher than the allowable 1–5 NTU. The trend is in line with the well‐documented results of riverine wetlands in the Himalayan foothills, where the monsoon runoff carries large amounts of suspended sediments on eroded slopes and disturbed catchment areas (Wulf et al. 2012). The level of DO was adequate at both sites across all seasons, which implies that there was sufficient photosynthetic activity and aeration and that neither site is oxygen‐depleted.

Calcium levels in Renuka Ji were also elevated in pre‐monsoon (94.092 mg/L) and post‐monsoon (93.2 mg/L), which aligns with the geological features of the watershed: calcareous rocks and soils are known to be the major contributors to calcium loading in the Himalayan water bodies (Das and Kaur 2008; Kumar et al. 2019), probably due to direct dissolution of calcium carbonate in the calcareous limestone geology of the Sirmaur region, coupled with the high rates of evapotranspiration and the concentrating effect of a closed lake basin during dry seasons (Kumar et al. 2019). This hypothesis is supported by the fact that the major decrease in the monsoon (66.96 mg/L) is due to the dilution of rainfall and slower weathering, which lowers dissolved calcium levels. The values of BOD and COD were within allowable limits all along, eliminating the possibility of heavy organic pollution at both sites.

### 4.2 Bacterial Diversity and Seasonal Trends

There was a lack of bacterial diversity at both sites and the culturable fraction of the microbial community was dominated by a relatively small number of genera. This high‐dominance, low‐diversity trend is a typical attribute of oligotrophic or nutrient‐restricted aquatic environments (Choden et al. 2022). NA, while widely used for general heterotrophic enumeration, is a comparatively nutrient‐rich medium that may under‐represent oligotrophic freshwater bacteria adapted to low‐nutrient conditions; a low‐nutrient medium such as R2A or a 10‐fold dilution of NA, would likely recover a broader range of culturable taxa in future sampling of these systems. In pure or semi‐pure Himalayan wetlands, the nutrient availability is more likely to be the main force behind bacterial abundance and diversity. Even those species that do settle are frequently adapted metabolically to the oligotrophic, cold and chemically unique environment.

Both sites had the highest pre‐monsoon counts. This is explained by increased temperatures in June, increased nutrient concentrations (reduced dilution) and stable hydrological conditions that favour bacterial growth. The CFU counts at both locations were reduced by the monsoon season, probably due to the dilution of bacterial populations by massive amounts of precipitation and the washout of surface‐associated microorganisms into the deep‐water column (Toraskar et al. 2022). In the Asan Conservation Reserve, the bacterial counts were found to recover in the post‐monsoon season (1.14 × 10^3^ CFU/mL), indicating that post‐monsoon stabilisation of the water column and nutrient enrichment by accumulated organic matter favour bacterial growth.

The occurrence of genera like *Pseudomonas, Agrobacterium, Acinetobacter* and *Acidovorax* at the locations is widely aligned with the reported bacterial communities of freshwater Himalayan systems (Ahmad et al. 2021; Kaushal et al. 2022). The presence of *Planococcus* and *Exiguobacterium* species, which were found in Asan during the post‐monsoon season, is attributed to their ability to survive in low‐temperature and nutrient‐depleted conditions, which is highly appropriate in Himalayan post‐monsoon conditions (Choden et al. 2022). The presence of *B. thuringiensis* (a sporing bacterium) in the post‐monsoon season also indicates the capacity of endospore‐formers to survive severe seasonal changes.

### 4.3 Absence of Bacterial Isolates at Renuka Ji During Post‐Monsoon

The fact that absence of culturable bacterial colonies was found at Renuka Ji within the tested dilution range (10^1^–10^4^) in the post‐monsoon season is an ecologically significant observation that should be interpreted with caution.

First, the physical and ecological processes are expected to have strong influences on the culturable bacterial dynamics, but they were not quantified in this study. Examples of this include the possibility of light attenuation with depth and competitive exclusion or metabolic partitioning, which are hypotheses yet to be tested and which will need to be validated in future through coupled optical and biogeochemical measurements. In addition, seasonal hydrological flux is an important parameter; in particular, the large increase in volume during and after the monsoon can result in a large dilution effect. The seasonal influx of rainfall can cause a reduction in the concentration of bacteria in the water column to near or below the lower limit of detection of standard serial dilution plating, which supplies a hydrologically based rationale for temporal variations in culturable bacterial recovery (Toraskar et al. 2022; Dhawde et al. 2018).

Second, in situ water temperature monitoring and high‐resolution chemical profiling were not carried out seasonally, but thermal and physical stresses are known to play an important role as filters in high‐altitude wetlands. Especially, the bacterial community undergoes a fraction that enters the viable‐but‐nonculturable (VBNC) state during the post‐monsoon environmental changes related to temperature drops, changes in chemical compositions and localised nutrient dilution (Liu et al. 2017; Pazos‐Rojas et al. 2023). VBNC bacteria are metabolically active and viable, but cannot grow on conventional growth media under conventional incubation conditions. Although the thermal and nutrient drivers are speculative for this baseline study because of the absence of continuous environmental sensor data, VBNC entry is a documented physiological mechanism that could explain the low counts of culturable microorganisms during the post‐monsoon season.

Third, although specific nutrient parameters like total nitrogen, phosphorus and total organic carbon were not measured in this study, the post‐monsoon oligotrophic condition of Renuka Ji lake is another possible factor. After post‐monsoonal flushing, the reduced nutrient availability may be limiting the metabolism of bacteria and biomass build‐up and may inhibit the ability of cells to grow into visible colonies on typical agar media within the standard incubation times (Choden et al. 2022). Therefore, the presence of taxa known to be oligotrophic suggests that nutrient limitation may be a likely potential selective factor in these waters, although this has to be tested. This hypothesis will need to be validated empirically with dedicated seasonal nutrient analyses. Importantly, these three mechanisms, physical hydrologic dilution, physiological entry into a VBNC state and oligotrophic growth suppression, are not mutually exclusive but are likely to act synergistically to explain the lack of culturable colonies over the tested dilution range.

### 4.4 Dominance by a Few Bacterial Species

The low bacterial diversity and domination of a few species found at both sites is indicative of a larger ecological trend in most oligotrophic or geochemically extreme freshwater systems. A plausible contributing factor, though not directly tested in this study, is competitive exclusion, whereby a small number of well‐adapted species outcompete others under resource‐limited conditions (Ghoul and Mitri 2016). The year‐long high EC values at Renuka Ji could cause selective pressure, favouring specifically adapted alkaliphilic or halotolerant bacteria, but not less tolerant taxa.

The pronounced monsoon turbidity at Asan may have limited photosynthetic nutrient production through light attenuation, though chlorophyll and primary productivity were not measured in this study and this mechanism remains speculative. The prevalence of genera like *Pseudomonas* and *Planococcus* in these locations could be due to their metabolic flexibility, biofilm‐forming ability and resistance to changing physicochemical conditions—characteristics that provide them with a competitive edge in these dynamic Himalayan wetlands (Choden et al. 2022).

### 4.5 Limitations and Future Outlook

Due to resource and time limitations, sampling was conducted in just 1 year and not repeated over several seasonal cycles. While these results are useful for short‐term seasonal variations, multi‐year monitoring is needed to ensure long‐term consistency and to observe year‐to‐year environmental fluctuations. One of the main drawbacks of the study was the culture conditions. This approach strongly selects for rapid‐growth and cold‐tolerant bacteria on nutrient‐rich media (NA) and biases against the native, cold‐adapted (psychrotolerant/psychrophilic) and oligotrophic bacterial communities intrinsic to Himalayan lacustrine ecosystems. Thus, the isolated taxa and reported counts are only a portion of the resident microbiome that is responsive to increased nutrient levels and temperature and is the culturable portion of the microbiome.

Future studies on high‐altitude Himalayan Ramsar wetlands should implement environmentally optimised culture strategies (e.g., use of low‐nutrient media such as R2A agar, reduced incubation temperatures 15°C–20°C and extended culture periods) and culture‐independent molecular assays (e.g., amplicon profiling of 16S rRNA) to account for all of the unculturable and cold‐adapted microbial diversity. Future research ought to use culture‐independent molecular techniques (metagenomics) to obtain a more holistic picture of the microbial community, including the VBNC fraction and ought to examine the functional roles of the newly reported isolates from these wetlands found here.

## 5 Conclusion

This paper presents the first combined seasonal evaluation of physicochemical and microbiological quality of Renuka Ji Lake and Asan Conservation Reserve, two Ramsar wetlands of the northwestern Himalaya. Several key findings emerge: both sites showed a high degree of seasonal variation in physicochemical parameters, with Renuka Ji having higher EC, especially during the pre‐monsoon and monsoon seasons. Despite lower ion levels, the Asan Conservation Reserve exhibited extreme monsoon turbidity, demonstrating the susceptibility of riverine wetlands to catchment erosion.

Molecular identification confirmed the presence of 11 bacterial isolates with NCBI GenBank accession numbers (PX980612.1‐PX980622.1); of these, *A. cavarae* is the isolate of particular interest, being comparatively less frequently reported from freshwater environments. Dilution, a possible shift to a VBNC state and reduced nutrient availability are plausible, nonexclusive explanations for the absence of culturable colonies recovered at Renuka Ji during post‐monsoon sampling, although none of these mechanisms was directly tested in this study. The culturable bacterial fraction recovered from both sites was dominated by a small number of environmentally adaptive genera; because no richness or diversity index was calculated and only morphologically distinct colonies were sequenced, this reflects the diversity of the recovered culturable morphotypes rather than the total bacterial community.

These results highlight the ecological sensitivity of Himalayan Ramsar wetlands and the significance of multi‐season monitoring structures. They represent a single annual cycle and should be interpreted as a seasonal snapshot; confirming that these patterns recur would require repeated sampling across multiple years.

## Author Contributions

**Sunil Puri:** investigation, supervision, writing – review and editing. **Shagufta Butt:** methodology, investigation, writing – original draft, data curation. **Sadanand Pandey:** supervision, writing – review and editing, resources. **Chandresh Kumari:** formal analysis, investigation, writing – review and editing. **Adane Woldemedhin Kalsido:** writing – review and editing, investigation, visualization, formal analysis. **Rakesh Kumar Shukla:** validation, visualization.

## Funding

The authors have nothing to report.

## Consent

The authors have nothing to report.

## Conflicts of Interest

The authors declare no conflicts of interest.

## Supporting information


**Figure S1:** Chromosomal DNA isolation workflow. The process encompasses three stages: bacterial growth and harvesting, cell lysis and DNA purification.
**Table S1:** Physicochemical properties of water samples during pre‐monsoon at Renuka Ji (Site 1) and Asan Conservation Reserve (Site 2).
**Table S2:** Physicochemical properties of water samples during monsoon at Renuka Ji (Site 1) and Asan Conservation Reserve (Site 2).
**Table S3:** Physicochemical properties of water samples during post‐monsoon at Renuka Ji (Site 1) and Asan Conservation Reserve (Site 2).
**Table S4:** BLAST identification and taxonomic assignment of bacterial isolates based on GenBank Accession and sequence similarity.

## Data Availability

The datasets generated and analysed during the current study are available from the corresponding author upon reasonable request. All relevant data supporting the findings of this study are included in the article and its Supporting Information.
