# Supplementary material for: Seasonal Baseline Evaluation of Physicochemical Parameters and Culturable Bacterial Characteristics in Northwestern Himalayan Ramsar Sites
Source: Environ Microbiol Rep. 2026 Sep 1;18(5):e70409. doi: 10.1111/1758-2229.70409 (PMC13533594; doi:10.1111/1758-2229.70409)
Supplement: Supplementary file 1 [file EMI4-18-e70409-s001.docx]

**Supplementary File**

**Seasonal Baseline Evaluation of Physicochemical Parameters and Culturable Bacterial Characteristics in Northwestern Himalayan Ramsar Sites**

**Shagufta Butt^1^, Chandresh Kumari^2^, Rakesh Kumar Shukla^3^, Sunil Puri^1^, Sadanand Pandey^2*^, Adane Woldemedhin Kalsido^4*^**

*^1^School of Biological & Environmental Sciences, Shoolini University, Solan 173229, Himachal Pradesh, India*

*^2^Faculty of Applied Sciences and Biotechnology, Shoolini University, Solan 173229, Himachal Pradesh, India*

*^3^School of Physics and Materials Science, Faculty of Science, Shoolini University, Solan 173229, Himachal Pradesh, India*

*^4^Department of Hydraulic and Water Resources Engineering, College of Engineering and Technology, Wachemo University, Ethiopia. P.O.Box:667*

*Corresponding author:* [*Sadanand.au@gmail.com*](mailto:Sadanand.au@gmail.com) (S.P); [adane.kalsido@wcu.edu.et](mailto:adane.kalsido@wcu.edu.et) (A.W.K)

***Figure S1:*** *Chromosomal DNA isolation workflow. The process encompasses three stages: bacterial growth and harvesting, cell lysis, and DNA purification.*

***Table S1.*** *Physicochemical properties of water samples during pre-monsoon at Renuka Ji (Site 1) and Asan Conservation Reserve (Site 2).*

| **S.No.** | **Parameters** | **Renuka Ji (Site 1)** | **Asan Conservation Reserve (Site 2)** | **Permissible Limit** |
| --- | --- | --- | --- | --- |
| 1 | pH | 8.28 ± 0.021 | 6.53 ± 0.197 | 6.5 to 8.5 **{CPCB, Class D (Propagation of wildlife and fisheries), 2012}** |
| 2 | Electrical Conductivity | 627.5 µS/cm ± 23.33 | 123.55 µS/cm ± 65.76 | <1000µS/cm (**CPCB, Class D guideline)** |
| 3 | Turbidity | 4 NTU ± 1.414 | 21.95 NTU ± 1.697 | 1-5 NTU {**BIS IS (Bureau of Indian Standards, Indian Standard) 10500:2012}** |
| 4 | Total Dissolved Solids | 315 mg/l ± 14.14 | 110 mg/l ± 7.071 | 500 mg/l (**CPCB, Class A)** |
| 5 | Total Hardness | 303 mg/l ± 1.414 | 54 mg/l ± 11.31 | 200-600 mg/l **(BIS IS 10500:2012)** |
| 6 | Dissolved Oxygen | 10 mg/l ± 2.687 | 10.1 mg/l ± 0.282 | $\geq$ 4.0 mg/l {**CPCB, Class D (Propagation of wildlife and fisheries), 2012}** |
| 7 | Biochemical Oxygen Demand | 10.65 mg/l ± 4.737 | 12.3 mg/l ± 5.232 | 30 mg/l **{CPCB General Standards for Treated Effluent Discharge into Inland Surface Waters, under the Environment Protection Rules, 1986 (General Statutory Rules 801(E)}** |
| 8 | Chemical Oxygen Demand | 117.3 mg/l ± 11.73 | 134.65 mg/l ± 70.21 | 250 mg/l **{CPCB General Standards for Treated Effluent Discharge into Inland Surface Waters, under the Environment Protection Rules, 1986 (General Statutory Rules 801(E)}** |
| 9 | Calcium | 94.092 mg/l ± 6.915 | 61.46 mg/l ± 43.82 | 75-200 mg/l **(BIS IS 10500:2012)** |
| 10 | Chloride | 5.395 mg/l ± 3.273 | 3.34 mg/l ± 2.545 | 250 mg/l (**CPCB, Class A/C)** |

*Note:* $\pm$ *means Standard Deviation (SD)*

***Table S2.*** *Physicochemical properties of water samples during monsoon at Renuka Ji (Site 1) and Asan Conservation Reserve (Site 2).*

| **S.No.** | **Parameters** | **Renuka Ji (Site 1)** | **Asan Conservation Reserve (Site 2)** | **Permissible Limit** |
| --- | --- | --- | --- | --- |
| 1 | pH | 8.55 ± 0.056 | 7.99 ± 0.014 | 6.5 to 8.5 **{CPCB, Class D (Propagation of wildlife and fisheries), 2012}** |
| 2 | Electrical Conductivity | 525.5 µS/cm ± 0.707 | 153.85 µS/cm ± 0.212 | <1000µS/cm (**CPCB, Class D guideline)** |
| 3 | Turbidity | 5 NTU ± 1.414 | 197 NTU ± 10.11 | 1-5 NTU {**BIS IS (Bureau of Indian Standards, Indian Standard) 10500:2012}** |
| 4 | Total Dissolved Solids | 269 mg/l ± 1.414 | 80 mg/l ± 0 | 500 mg/l (**CPCB, Class A)** |
| 5 | Total Hardness | 247 mg/l ± 4.242 | 66 mg/l ± 2.828 | 200-600 mg/l **(BIS IS 10500:2012)** |
| 6 | Dissolved Oxygen | 6.65 mg/l ± 1.909 | 6.05 mg/l ± 0.919 | $\geq$ 4.0 mg/l {**CPCB, Class D (Propagation of wildlife and fisheries), 2012}** |
| 7 | Biochemical Oxygen Demand | 14 mg/l ± 1.414 | 9 mg/l ± 1.414 | 30 mg/l **{CPCB General Standards for Treated Effluent Discharge into Inland Surface Waters, under the Environment Protection Rules, 1986 (General Statutory Rules 801(E)}** |
| 8 | Chemical Oxygen Demand | 144.8 mg/l ± 15.83 | 152.65 mg/l ± 13.647 | 250 mg/l **{CPCB General Standards for Treated Effluent Discharge into Inland Surface Waters, under the Environment Protection Rules, 1986 (General Statutory Rules 801(E)}** |
| 9 | Calcium | 66.96 mg/l ± 1.463 | 46.61 mg/l ± 4.256 | 75-200 mg/l **(BIS IS 10500:2012)** |
| 10 | Chloride | 5.44 mg/l ± 3.471 | 2.24 mg/l ± 0.360 | 250 mg/l (**CPCB, Class A/C)** |

***Table S3.*** *Physicochemical properties of water samples during post-monsoon at Renuka Ji (Site 1) and Asan Conservation Reserve (Site 2).*

| **S.No.** | **Parameters** | **Renuka Ji (Site 1)** | **Asan Conservation Reserve (Site 2)** | **Permissible Limit** |
| --- | --- | --- | --- | --- |
| 1 | pH | 8.33 ± 0.070 | 6.54 ± 0.452 | 6.5 to 8.5 **{CPCB, Class D (Propagation of wildlife and fisheries), 2012}** |
| 2 | Electrical Conductivity | 425 µS/cm ± 35.35 | 134.25 µS/cm ± 10.96 | <1000µS/cm (**CPCB, Class D guideline)** |
| 3 | Turbidity | 5 NTU ± 1.414 | 16.7 NTU ± 2.404 | 1-5 NTU {**BIS IS (Bureau of Indian Standards, Indian Standard) 10500:2012}** |
| 4 | Total Dissolved Solids | 302.5 mg/l ± 17.67 | 105 mg/l ± 21.21 | 500 mg/l (**CPCB, Class A)** |
| 5 | Total Hardness | 291 mg/l ± 7.071 | 45 mg/l ± 4.242 | 200-600 mg/l **(BIS IS 10500:2012)** |
| 6 | Dissolved Oxygen | 9.7 mg/l ± 4.666 | 9.7 mg/l ± 1.838 | $\geq$ 4.0 mg/l {**CPCB, Class D (Propagation of wildlife and fisheries), 2012}** |
| 7 | Biochemical Oxygen Demand | 16.95 mg/l ± 3.323 | 19.95 mg/l ± 11.808 | 30 mg/l **{CPCB General Standards for Treated Effluent Discharge into Inland Surface Waters, under the Environment Protection Rules, 1986 (General Statutory Rules 801(E)}** |
| 8 | Chemical Oxygen Demand | 104.8 mg/l ± 10.182 | 92.95 mg/l ± 2.333 | 250 mg/l **{CPCB General Standards for Treated Effluent Discharge into Inland Surface Waters, under the Environment Protection Rules, 1986 (General Statutory Rules 801(E)}** |
| 9 | Calcium | 93.2 mg/l ± 0 | 39.31 mg/l ± 2.206 | 75-200 mg/l **(BIS IS 10500:2012)** |
| 10 | Chloride | 4.10 mg/l ± 2.906 | 1.53 mg/l ± 0.728 | 250 mg/l (**CPCB, Class A/C)** |

***Table S4.*** *BLAST identification and taxonomic assignment of bacterial isolates based on GenBank Accession and sequence similarity*

| **Isolate Code** | **Top Match (Type Strain)** | **GenBank Accession** | **Query Coverage (%)** | **Percentage Identity (%)** | **E-value** | **Assigned Taxonomy** |
| --- | --- | --- | --- | --- | --- | --- |
| R(SP) 1.3 | *Comamonas*  20-RIF-16 | PX980613.1 | 100% | 100% | 0.0 | *Comamonas* sp. |
| R(SP) 2.4 | *Fictibacillus*  AK 57 | PX980612.1 | 100% | 100% | 0.0 | *Fictibacillus* sp. |
| R(MP) 3.1 | *Endophytic Bacterium*  GGR | PQ001997.1 | 100% | 100% | 0.0 | *Endophytic bacterium* |
| A(MP) 2 | *Pseudomonas* PA35 | KT710797.1 | 100% | 99.85% | 0.0 | *Pseudomonas* sp. |
| R(MP) 4 | *Acidovorax*  C9-1 | PX980615.1 | 100% | 100% | 0.0 | *Acidovorax* sp. |
| A(SP) 1 | *Agrobacterium cavarae* CEMTC_11674 | PX925985.1 | 100% | 100% | 0.0 | *Agrobacterium cavarae* |
| A(SP)1.2 | *Planococcus rifietoensis* CEMTC_7114 | PV538487.1 | 100% | 99.56% | 0.0 | *Planococcus rifietoensis* |
| A(SP)1 | *Exiguobacterium undae* CEMTC_9647 | PP294643.1 | 100% | 100% | 0.0 | *Exiguobacterium undae* |
| A(SP) 1.1 | *Exiguobacterium undae*  SW19 | OQ186877.1 | 100% | 100% | 0.0 | *Exiguobacterium undae* |
| A(MP) 2 | *Planococcus ruber* CD8 | PX980620.1 | 100% | 100% | 0.0 | *Planococcus ruber* |
| A(SP)1.3 | *Bacillus thuringiensis*  S8-3_EP | PX980622.1 | 100% | 95% | 0.0 | *Bacillus thuringiensis* |

*Note: The first-ranked match was recently updated based on our FASTA sequence results. As the NCBI database is frequently updated with new sequence submissions and annotations, the results may differ from those reported in the manuscript and the present table*
